# Effectiveness of Digital Cognitive Behavior Therapy for the Treatment of Insomnia: Spillover Effects of dCBT

**DOI:** 10.3390/ijerph19159544

**Published:** 2022-08-03

**Authors:** Xinyi Li, Hongying Liu, Ming Kuang, Haijiang Li, Wen He, Junlong Luo

**Affiliations:** 1 Department of Psychology, Educational College, Shanghai Normal University, Shanghai 200234, China; zslxydyxa@163.com (X.L.); haijiangli@shnu.edu.cn (H.L.); 2 Hangzhou Kang Sheng Health Consulting Co., Ltd., Hangzhou 310023, China; hongyingliu@91jkys.com (H.L.); mkuang2@91jkys.com (M.K.)

**Keywords:** digital cognitive behavior therapy, insomnia, fatigue, flow, cognitive flexibility, spill-over effect

## Abstract

The effects of digital Cognitive Behavior Therapy for insomnia (dCBT-i) on sleep quality have been previously demonstrated but the spillover effects on fatigue, flow (a state of immersion in activities of interest), and cognitive flexibility remain unclear. The current study examined the effectiveness of dCBT-i. A total of 97 college students (20.96 ± 1.87 years, 73.1% female students) were randomly selected from a shortlist and divided into sleep intervention (*n* = 39), conventional education (*n* = 37), and healthy control (*n* = 21) groups. Task switching paradigm, Fatigue Severity Scale (FSS), Flow Experience Scale (FES), and the Chinese version of the Pittsburgh Sleep Quality Index (CPSQI) were measured pre- and post-intervention. Results show that the sleep quality of the intervention group improved, and fatigue was relieved. Participants in the sleep intervention group had increased flow experience scores post-intervention and improved cognitive flexibility. The control group’s sleep quality deteriorated and fatigue level increased. dCBT-i can not only achieve a significant improvement in sleep quality and reduce fatigue, but also improve learning abilities, quality of life, flow, and cognitive flexibility. Future research should pay attention to indicators such as work efficiency, sedative use, and the durability and stability of such effects.

## 1. Introduction

Insomnia is the subjective dissatisfaction with the quantity or quality of one’s sleep. It is a psychophysiological condition caused by sensory and information processing abnormalities, attentional bias, or end-organ function abnormalities. Insomnia often manifests itself as difficulty falling asleep, maintaining sleep, or waking up early, causing significant stress and impairment of functions, such as remembering, decision making, and walking [1,2,3]. Approximately 5–15% of adults meet the formal diagnostic criteria for chronic insomnia (now known as insomnia disorder), 25% have insomnia symptoms, and 33% report dissatisfaction with sleep [4,5]. However, only 13% of people with insomnia have ever consulted a healthcare provider for sleep difficulties [6]. Insomnia is associated with physical and mental comorbidities, such as anxiety, depression, and diabetes [4], while poor sleep quality is often accompanied by symptoms such as daytime fatigue, cognitive impairment, or mood disorders [7,8]. Moreover, it has a negative impact on daily learning, work efficiency, and happiness [9]. Considering these factors, insomnia may affect the quality of one’s life.

### 1.1. Digital Cognitive Behavioral Therapy for Insomnia

As face-to-face treatment and consultation for insomnia consume substantial time and energy, and physician resources are not always sufficient [5,10,11,12], digital Cognitive Behavioral Therapy for insomnia (dCBT-i) is considered an effective treatment that can cater to the needs of a larger population. As its name suggests, dCBT-i improves and treats insomnia in two distinct aspects, cognition and behavior, and contains a variety of components including sleep hygiene education, stimulus control, sleep restriction, relaxation, and cognitive therapy (Table 1). However, it differs from face-to-face therapy in that all the interventions are delivered online, such as through mobile phone software, web guidance, WeChat program, telephone calls, and online meetings [4,12]. Rajabi Majd et al. [13], through a randomized controlled trial, found that dCBT-i improved insomnia symptoms, depression, and anxiety in patients, and this effect persisted for six months after the intervention. An experiment conducted during the COVID-19 pandemic also demonstrated that dCBT-i can reduce insomnia and increase health resilience [14].

The flexibility and accessibility of dCBT-i make it convenient, efficient, and inexpensive, and people can even use the software applications themselves. It has been shown to be effective in improving sleep conditions and is recommended as an initial treatment for insomnia in adults [9,15,16].

### 1.2. Fatigue and Insomnia

Insomnia is associated with significant healthcare costs, reduced productivity, and diminished health-related quality of life [17,18]. Among these health-related factors, fatigue is considered characteristic of insomnia [7] and associated with subjective well-being in daily life. It is an important indicator of health literacy and health risk behavior [19], which are significant for college students. Fatigue is defined as reversible physical or cognitive impairment caused by mental or physical activity, diet, or illness, manifested as a lack of motivation to do things and a desire to rest [20]. Insomnia is often shown to be comorbid with unexplained fatigue [21]. Pastier and colleagues [22] found that physical and mental fatigue are associated with sleep quality in healthy adults. Multiple regression models have shown that sleep quality is influenced by state and trait of physical fatigue [23]. A meta-analysis showed that, when compared with a placebo, CBT-i significantly improved depressive symptoms in patients, but not fatigue and the high heterogeneity among the research indicated that further clinical study is needed to confirm the spillover effect of CBT-i on fatigue [24]. Moreover, there is currently limited research exploring the specific relationship between fatigue severity and poor sleep quality among college students.

### 1.3. Cognitive Flexibility

Previous studies on the relationship between insomnia and cognitive flexibility have been inconsistent. Killgore et al. showed that insufficient sleep can reduce the flexibility of thought but used questionnaires that are considered subjective indicators [25]. Killgore’s team also found comparable results with the Iowa Gambling Task, where sleep deprivation led to a decline in cognitive flexibility [26]. A published review suggested that sleep deprivation can severely impair cognitive flexibility; however, evidence in this regard has been less consistent [27]. A previous meta-analysis by Fortier-Brochu et al. [28] found that there were no significant differences in cognitive flexibility between healthy and insomnia populations. A possible explanation for these discrepancies could be that while poor sleep quality does affect people’s cognitive flexibility, it may not appear to be significant because of differences in experimental tasks. Herbert et al. [29] completed a meta-analysis pointing out the limited effects of CBT-i on measures of self-reported cognitive functioning, and few of the effects were statistically significant. On the other hand, the study also indicated the need for more effective and powered randomized controlled trials to understand the effects of CBT-i. Regarding the relationship between sleep and cognitive flexibility, research has produced varying results due to the differences between the scale method and objective measurement, the different experimental paradigms adopted in the study of cognitive flexibility, and the differences in experimental interventions. However, there are still few studies that have focused on the effects of CBT-i on objective cognitive performance [29].

### 1.4. Flow and Insomnia

Flow, also known as optimal experience, is defined as the state of extreme pleasure in which persons immerse themselves in an activity that deeply interests them, forgetting the passage of time [30]. In the state of flow, individuals tend to concentrate on the present, enjoy the activity process while having an ardent desire for control, understand where things are going, and process the ability to deal with challenges [31]. However, studies have shown that flow experience is reduced after sleep deprivation, that negative emotions increase, and psychomotor performance decreases [32]. Original research on flow focused on work, sports, musical performance, and games, and the results showed that participants in the flow state performed better in these areas [33,34,35]. In the state of insomnia or partial sleep deprivation, the physical activity indicators, sports performances, and emotion regulation of the participants all decreased [36]. One possible explanation is that insomnia affects the flow experience. Kaida et al. [37] found that sleepiness is significantly and negatively correlated with the flow experience. Smith et al. [38] determined that personality traits, state of flow, and situational characteristics in older adolescents influence their bedtimes. Previously, research on the relationship between flow and insomnia was limited, and there were some shortcomings including small sample sizes (to our knowledge, the sample sizes were all less than 17), inadequate criteria for the diagnosis of insomnia [38], different measurement methods for flow [37], and the experience of flow not being a main variable in the results. In an attempt to overcome these limitations, our research explores whether dCBT-i’s improvement of sleep quality results in an increase in the experience of flow.

### 1.5. The Current Study

The spillover effect is the term used to describe a condition in which an activity that is conducted not only produces the expected effect but also has an impact in other areas. The idea that an activity could have external benefits has come to be widely accepted in economics, management, and many psychological concepts [39,40]. Previous studies suggested that dCBT-i can improve functional health, psychological wellbeing, and sleep-related quality of life, including fatigue, the focus of this study [41]. In this paper, we seek to answer the question of whether dCBT-i can show spillover effects in relieving fatigue and improving flow and cognitive flexibility, in addition to relieving insomnia.

First, verify previous results, many studies have demonstrated the effectiveness of dCBT in the treatment of insomnia through rigorous experiments, larger samples, and cross-cultural experiments [3,4,5,6,7,8,9,10,11,12].

**Hypothesis** **1** **(H1).** *dCBT-i can effectively relieve insomnia and improve sleep conditions.*

Second, although fatigue is thought to be inversely related to sleep quality [22,23], the effect of dCBT-i on fatigue has not been adequately studied [24].

**Hypothesis** **2** **(H2).** *dCBT-i can alleviate subjective fatigue.*

Third, previous studies on insomnia and cognitive flexibility are not always consistent [25,26,27,28,29], but behavioral experimental results show that improved sleep can improve cognitive flexibility [26,27].

**Hypothesis** **3** **(H3).** *dCBT-i can enhance cognitive flexibility.*

Fourth, there are only a few small sample size studies demonstrating that insomnia reduces flow and thus affects people’s daily activities [36,37,38].

**Hypothesis** **4** **(H4).** *dCBT-i can improve the daily experience of flow.*

## 2. Materials and Methods

### 2.1. Design and Participants

The current study was a randomized controlled trial with three groups (i.e., intervention group vs. conventional education group vs. healthy controls) and two assessment time points (baseline and post-intervention), conducted online between September and November 2021. We included a healthy control group to rule out the effects of events affecting the entire cohort such as the recurrence of COVID-19 and the beginning of the university term. Participants were recruited from several universities in Shanghai and Guizhou. A total of 1698 students voluntarily participated in the study by scanning the Quick Response or QR code of the questionnaire through the WeChat mobile application and registered for the experiment.

**Figure 1:**
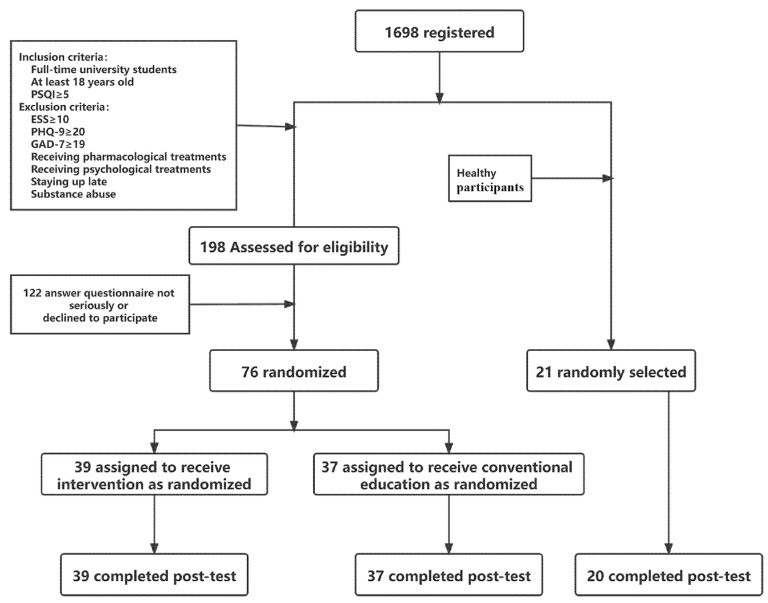
CONSORT flow diagram.

The inclusion criteria were as follows: (1) full-time college student and at least 18 years of age; and (2) Pittsburgh Sleep Quality Index (PSQI) score of 5 or higher, which indicates poor sleep quality [42]. The exclusion criteria were as follows: (1) Epworth Sleepiness Scale score of 10 or higher; (2) 9-item Patient Health Questionnaire score of 20 or higher or a 7-item Generalized Anxiety Disorder scale score of 19 or higher, which implies that insomnia may be caused by depression or anxiety [13,43]; (3) self-reported suicidal ideation with intent; (4) currently undergoing pharmacological/psychological treatments for insomnia; (5) report a diagnosis of any physical/psychiatric disease that causes insomnia (e.g., primary/secondary pain, nervous system damage, schizophrenia, etc.); and (6) substance abuse which may lead to insomnia (e.g., caffeine, nicotine, alcohol, etc.). Ultimately, 198 college students met the experimental criteria. Among these, 122 participants were excluded because they were unwilling to participate in the follow-up, or the time they had taken to answer the questionnaire was too short, indicating that they may not have answered genuinely. Therefore, 76 participants with sleep problems were finally included in the current study. They were randomly divided into two groups: sleep intervention (*n* = 39) and conventional education (*n* = 37). We randomly selected 21 participants who had PSQI scores less than 5 [42], as the healthy control group (i.e., the 21 participants did not have any insomnia-related clinical symptoms or possible comorbidities and were considered healthy for the purpose of this study). Figure 1 shows the participant selection flowchart. There were no significant differences in age, sex, or annual household income between the three groups of participants (*p* ≥ 0.166). The study was approved by the local ethics committee of Shanghai Normal University and all participants signed consent forms at the beginning of the research. The trial was registered at Chinese Clinical Trial Registry before commencement (Registration ID: ChiCTR2100053172). After completion of the post-intervention questionnaires, each participant in the intervention, conventional education, and healthy control groups received 200 yuan, 150 yuan and 60 yuan, respectively, as monetary compensation through WeChat payment.

### 2.2. Procedure and Materials

We collected baseline responses to the questionnaires and randomly divided eligible participants (*n* = 76) into sleep intervention (*n* = 39) and conventional education (*n* = 37) groups. We then randomly selected healthy controls (*n* = 21). Participants were first asked to complete a task switching exercise online to measure cognitive flexibility. Subsequently, the three groups completed a three-week period of Cognitive Behavioral Therapy (CBT) intervention, conventional sleep education, and no manipulation, respectively. Finally, all participants completed a post-test questionnaire (which included the PSQI, Fatigue Severity Scale (FSS), and Flow Experience Scale (FES)) and task switching exercises online, with a reminder that they had to be completed within five days. We also recorded their login times and task completion rates to ensure the effectiveness of the intervention. 

#### 2.2.1. CBT Intervention and Conventional Sleep Education

For the three-week dCBT-i, WeChat we used to conduct core courses once a day, each of about 10–15 min duration, which included sleep hygiene education (week 1) (e.g., symptoms of insomnia, treatment, how to assess sleep condition and select tasks); sleep restriction, stimulation control, regular exercise, relaxation training, and control intake (week 2); gratitude journal, cognitive reconstruction, correct thoughts about sleeping medicine, and relaxation of the mind (week 3) [44]. After the first login, the system recorded participants’ login history every day. Participants were required to complete the videos, punch in, and maintain sleep diaries every day. They were reminded to complete the tasks on the dCBT-i applet of WeChat during the intervention. The system also recorded the video playback progress and punch-in status. The daily sleep diary recorded their bedtime, time of falling asleep, number of times of waking up during the night, wake-up time, daytime naps, and intake of tobacco, alcohol, and caffeine.

Conventional sleep education provided fixed articles on how to develop a regular routine, the importance of sports, and basic lifestyle, environmental, and behavioral recommendations that might help improve sleep (i.e., sleep hygiene education, the content which was presented as videos of core courses in the first week of CBT intervention was presented in the form of an essay to the second group in the trial), and each article took approximately three minutes to read. Participants were required to read the article posted on the WeChat official account every day and the number of readings for each article was recorded. As with the intervention group, conventional sleep education lasted 3 weeks. There are both similarities and differences between the patient education and dCBT-i programs. The content of the articles presented to the education group was contained within the CBT videos; however, the CBT program intervened through online tools and weekly interactive tasks, while the patient education program did so through electronic articles. In theory, CBT intervention includes complete cognitive to behavioral guidelines by professionals and provides questions with their answers, while conventional sleep education provides popular scientific versions of sleep knowledge without restricting behavior or changing beliefs. In terms of time investment, participants in the CBT intervention group needed to invest 10–15 min or more every day paying attention to their body and adopting behaviors beneficial to sleep and so on, while the conventional sleep education group only spent three minutes in reading an article. Participants were encouraged to contact the research team if they experienced any adverse events, had any questions, experienced any problems related to their health, or faced technical issues. dCBT-i is regarded as an intervention with minimal adverse effects, and none have been reported in previous large-scale trials [41].

#### 2.2.2. Measurement of Outcomes

**Figure 2:**
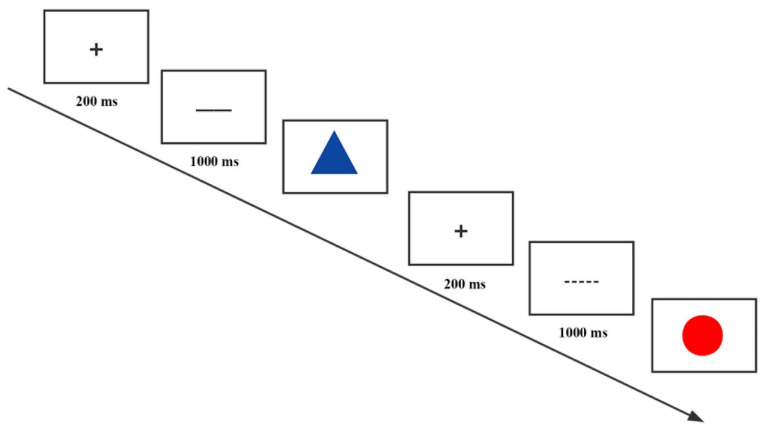
Flow chart of task switching paradigm. A “+” fixation was presented in the center of the computer screen for a duration of 200 ms, followed by a 1000 ms cue trail and a target stimulus presentation. The target stimulus had no time limit, and participants’ responses will be recorded.

- *Cognitive flexibility tasks:* At present, the study of cognitive flexibility focuses on two paradigms: deductive and inductive. The main deductive paradigm assessment methods are the Dimensional Change Card Sort, Stroop, Stop-Signal Task, Hand game, Ramp Causality Task, and day and night task. Inductive paradigm methods include the Wisconsin Card Sorting Test, Flexible Induction of Meaning Task, and Flexible Item Selection Task [45]. We used the task switching test to assess the cognitive flexibility of all groups of participants. A testing website was used to present the assessment program on participants’ computers, who were instructed to adjust the resolution. The assessment consisted of cue and target stimuli. There were two kinds of cues—solid lines and dashed lines. The target stimulus contained two dimensions of color (red/blue) and shape (circle/triangle), and the subset of the two dimensions was randomly combined to form four target stimuli: red triangle, red circle, blue triangle, and blue circle. The cue and target stimuli were presented at random. The task required participants to react to the target stimulus (shape/color) based on the cues (solid/dashed lines) such as “react to the shape when the cue is a solid line (press the “F” key for triangles and press the “J” key on the circle)”; “react to the color when the cue is a dashed line (press the “F” key in blue, press the “J” key in red).” If the present cue is the same as the previous cue, it is called a task repetition, and if it is different from the previous cue, it is called a task switch. The tasks appeared at random. In the test, a “+” fixation was presented in the center of the computer screen for a duration of 200 ms, followed by a 1000 ms cue trail and a target stimulus presentation. The target stimulus had no time limit, and the computer automatically recorded participants’ response time and accuracy. The experiment consisted of 20 practice and 120 formal trials, with feedback on whether the practice phase was correct or not in the formal lab phase. The program flow is illustrated in Figure 2. The accuracy rate of the repeat and switch tasks, and the reaction time were used as analysis indicators.
- *Pittsburgh Sleep Quality Index (PSQI)*: The Chinese version of the PSQI (CPSQI), a widely used quantitative scale with good reliability and validity, was used to assess participants’ sleep status. The PSQI was originally designed to measure sleep quality in clinical populations [46]. The use of the scale was then extended to people with insomnia and healthy populations [47].
- The CPSQI, revised by previous investigators, has an overall reliability coefficient of 0.82–0.83 [48]. It includes 19 questions, divided into seven dimensions: (1) sleep quality; (2) sleep time; (3) sleep duration; (4) sleep efficiency; (5) sleep disorders; (6) use of hypnotics; and (7) daytime dysfunction. Each dimension was scored from 0 to 3 and the scores were added to obtain the total CPSQI score; a higher score indicated poorer subjective sleep quality. A CPSQI score ≥ 5 was used as the truncated value to determine sleep quality [39].
- *Fatigue Severity Scale (FSS)*: The degree of fatigue was measured using the FSS. It is a self-assessment scale used to evaluate fatigue severity and its impact on daily functioning; it contains nine questions on a seven-point scale (1 = total disagreement, 7 = complete agreement). The higher the score, the more severe the fatigue level. The FSS was originally compiled by Krupp et al. [49] to measure the degree of fatigue in patients with multiple sclerosis. Studies have proven that it has good reliability and validity in other populations, with Cronbach Alpha > 0.89 [50,51].
- *Flow Experience Scale* (*FES*: Hoffman and Novak [52] were the first to propose a one-dimensional flow structure and develop a flow experience scale. In addition, later studies have proposed a two-dimensional theory of flow (enjoyment and concentration) and created a scale accordingly [53]. Some researchers are of the opinion that flow can be measured in four dimensions: enjoyment, concentration, control, and curiosity [54]. This study uses a localized, single-dimensional scale (the FES) to measure flow, and considers the loss of sense of time, pleasure, control, and concentration [55]. We adapted this scale from its predecessor and combined it with daily learning to produce a total of five items on a Likert-scale ranging from 1 (total disagreement) to 7 (complete agreement) (e.g., When I am studying, I sometimes ignore what is happening around me). The FES used in this experiment has high reliability and validity (Cronbach Alpha = 0.850).

### 2.3. Analysis

A mixed ANOVA was conducted on all scales (the CPSQI, FSS, and FES) and cognitive flexibility outcomes, with one within-subject (time effect: baseline vs. post-intervention) and one between-subject (group effect: intervention group vs. conventional education group vs. healthy controls). For those with a significant interaction effect (group × time), a simple effect analysis was performed. The change in score was calculated as the difference between the pre- and post-test scores. Spearman correlations between the change in scores and demographic data were performed. The effect size of repeated measures ANOVA was denoted by $\eta_{p}^{2}$. Simple effect Cohen *ds* were calculated on the difference in change scores divided by the pooled standard deviation of the change. A Cohen’s *d* of 0.20 indicates a small, 0.50 a moderate, and 0.80 a large effect. Statistical significance was set at *p* = 0.05.

## 3. Results

**Figure 3:**
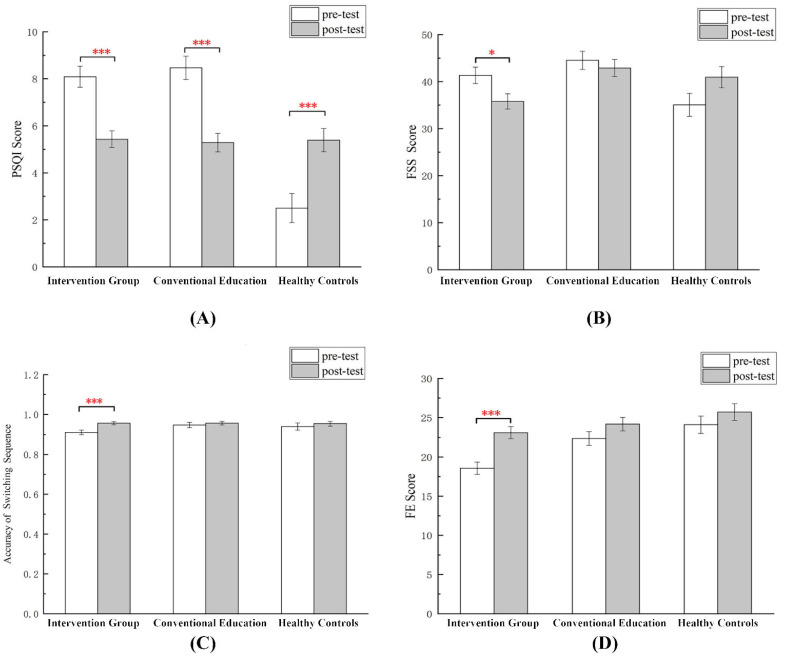
Means with standard error bars for changes in (**A**) PSQI score; (**B**) FSS score; (**C**) switch tasks accuracy; (**D**) FES score from baseline to follow-up between the intervention, conventional education, and healthy control groups. * *p* < 0.05; *** *p* < 0.001.

As one healthy control participant dropped out at the post-test stage, only 96 participants were included in this analysis. Table 2 shows the demographic data of the participants who were 20.96 years old on average, were mostly female (76.3%), were mostly pursuing a bachelor’s degree (68.0%), and their families earned 50,000–100,000 RMB annually (29.9%). There was no significant difference between the dCBT-i sleep intervention and conventional education groups, except for flow [t(74) = −3.232, *p* = 0.002] at baseline. This interaction is illustrated in Figure 3.

### 3.1. CPSQI Differences

For the CPSQI score, there was a significant time effect [*F*(1, 93) = 10.733, *p* < 0.001, $\eta_{p}^{2}$ = 0.103], group effect [*F*(2, 93) = 17.331, *p* < 0.001, $\eta_{p}^{2}$ = 0.272], and group × time effect [*F*(2, 93) = 31.259, *p* < 0.001, $\eta_{p}^{2}$ = 0.402]) (Figure 3A).

Further, simple effect analysis revealed that the CPSQI score (before: *M* = 8.086, *SE* = 0.445; after: *M* = 5.429, *SE* = 0.353; *p* < 0.001) in the experimental group decreased significantly (Cohen’s *d* = −1.030, 95% CI = [−1.698, −0.362]). Similarly, in the conventional education group, the PSQI score decreased significantly (before: *M* = 8.464, *SE* = 0.497; after: *M* = 5.286, *SE* = 0.395; *p* < 0.001) from baseline to follow-up (Cohen’s *d* = −1.355, 95% CI = [−2.070, −0.641]). As for healthy controls, the CPSQI score significantly increased (before: *M* = 2.500, *SE* = 0.620; after: *M* = 5.389, *SE* = 0.493; *p* < 0.001) (Cohen’s *d* = 1.491, 95% CI = [0.500, 2.482]).

### 3.2. FSS Differences

FSS scores had a significant effect on the group [*F*(2, 93) = 4.022, *p* = 0.021, $\eta_{p}^{2}$ = 0.080] and time × group interaction [*F*(2, 93) = 6.258, *p* < 0.001, $\eta_{p}^{2}$ = 0.170]. There was no time effect on the FSS (Figure 3B).

Simple effect analysis showed that in the intervention group, FSS decreased significantly (before: *M* = 41.343, *SE* = 1.748; after: *M* = 35.800, *SE* = 1.617; *p* = 0.018) (Cohen’s *d* = −0.561, 95% CI = [−1.201, −0.079]). As for the healthy controls, the increase in FSS score reached marginally significant levels (before: *M* = 35.056, *SE* = 2.438; after: *M* = 40.944, *SE* = 2.255; *p* = 0.069) (Cohen’s *d* = 0.786, 95% CI = [−0.124, 1.696]).

There was no time effect on the FSS, which meant that although the fatigue of the intervention group was relieved, the fatigue of the traditional education group was not relieved, and the fatigue of the healthy control group even increased after the experiment. This demonstrates the spillover effect of dCBT-i on fatigue.

### 3.3. Cognitive Flexibility Differences

We conducted an ANOVA to determine the difference in cognitive flexibility between the groups and times, and the results are shown in Figure 3C. As predicted, we found a significant group × time effect for switch task accuracy [*F*(2, 77) = 3.497, *p* = 0.035, $\eta_{p}^{2}$ = 0.083]. No significant differences were found for the rest of the markers of cognitive flexibility.

Further, simple effect analysis revealed that before and after the intervention, the accuracy of the switch task (before: *M* = 0.911, *SE* = 0.012; after: *M* = 0.956, *SE* = 0.008; *p* < 0.001) in the experimental group increased significantly (Cohen’s *d* = 0.069, 95% CI = [−0.559, 0.697]).

### 3.4. FES Differences

There was a significant time effect [*F* (1, 93) = 11.682, *p* = 0.001, $\eta_{p}^{2}$ = 0.112] and group effect [*F*(2, 93) = 9.519, *p* = 0.003, $\eta_{p}^{2}$ = 0.119], and a marginally significant group × time effect [*F*(2, 93) = 2.832, *p* = 0.064, $\eta_{p}^{2}$ = 0.057] for FES scores (Figure 3D).

A simple effect analysis found that the flow experience score (before: *M* = 18.692, *SE* = 0.787; after: *M* = 23.231, *SE* = 0.768; *p* < 0.001) in the experimental group increased significantly (Cohen’s *d* = 1.214, 95% CI = [0.531, 1.897]).

## 4. Discussion

Our research provides important insight into insomnia, and the mental difficulties it can trigger, and shows that dCBT-i can effectively intervene in its management. dCBT-i was also found to have a positive impact on other aspects, i.e., a spillover effect.

In this study, we had three groups (intervention group vs. conventional education group vs. healthy controls) and two assessment time points (baseline and post-intervention). In the three-week intervention, sleep quality in the intervention and conventional education groups improved significantly, indicating that the dCBT-i applet of the WeChat intervention could improve sleep quality and treat insomnia, which is consistent with previous studies that verified its effectiveness [13,56,57]. Furthermore, the CPSQI score in the control group increased significantly, suggesting poor sleep quality. One explanation for this is that the participants were college students who were beginning school; changes in the living environment and other maladjustment factors could be important factors for the deterioration of sleep quality. This may also be due to the COVID-19 pandemic, which has led to increased anxiety and insomnia [16]. First, research shows that people’s insomnia and fatigue increased during the pandemic [58,59,60], and approximately 60% of people reported increased insomnia symptoms, representing a tripling of risk, during this period [61,62,63]. Brown et al. [64] demonstrated that worrying about the outbreak, rather than exposure to risk factors for the virus, causes increased insomnia. A large-sample study shows that the excessive use of online media during a pandemic causes both physical and psychological fatigue in people [60]. Second, negative emotions brought about by the pandemic affected students’ sleep quality. During the pandemic, students’ worries about the future caused more anxiety, depression, and other troubling emotions [65]. Pandemics can significantly increase the general public’s depression, anxiety, and stress levels [66]. Students spent more time in relatively crowded dormitories rather than at libraries with a good learning environment. This was not conducive to disease prevention and control, causing more anxiety, irritability, and other negative emotions. College students were more likely to get all kinds of negative news from their frequent use of mobile phones [65,67]. Third, switching to the online mode increased working people’s burden because people need to learn and adapt to online office software and processes [68], and added to students’ academic burden. Students experienced high levels of academic stress [69]. Online teaching was adopted in courses to reduce the risk of close physical contact.

It must be noted that there may exist additional variables such as the placebo effect. The intervention group and conventional education group knew that they were in a sleep experiment and undertook activities to benefit sleep. Under this condition, the sleep status of the intervention group improved enough to explain the reliability of the dCBT-i. The decline in insomnia in the conventional education group indicates that reading sleep hygiene education articles can also improve insomnia, which may be attributed to the placebo effect. Therefore, the advantages of dCBT-i may be better reflected in other aspects such as flow, fatigue, and cognitive flexibility, that is, the spillover effect.

Multitasking is important in all aspects of daily life. Only the intervention group showed a significant increase in the accuracy of the switch task, indicating that dCBT-i does improve the cognitive flexibility of participants. As a more convincing indicator of cognitive flexibility, the switch task indicates the fluency and accuracy of task switching [70,71]. The task switching cost of the intervention group was reduced with the increase in accuracy of the switching sequence. The significant increase in accuracy in the intervention group was inseparable from the intervention. Unfortunately, no significant differences between the three groups were found in other markers, either before or after the intervention. It is likely that changes in cognitive function were initially reflected to a greater extent in brain function, and behavioral indicators were difficult to capture. Future experiments should consider using electroencephalograms to explore the differences in cognitive flexibility between patients with and without insomnia as well as changes in brain function before and after intervention.

Moreover, in the intervention group, a significant decrease in fatigue and increase in flow also reflected the spillover effect of dCBT-i. Specifically, fatigue in the conventional education group was also relieved but did not reach a significant level. Although the fatigue level of healthy controls increased, it did not reach a significant level. Changes in the fatigue levels of healthy people, as mentioned above, are likely to stem from the COVID-19 crisis, academic pressure, and other factors. This is similar to the results of previous studies [44,72,73]. Another earlier research with a large sample size (*n* = 1721) showed that dCBT-i reduced daytime fatigue and improved other indicators of health and happiness, providing further evidence for the stability of this effect [44]. Although there were differences in flow among the three groups at baseline [*F*(94) = 11.721, *p* < 0.001], the intervention group had the lowest flow level at the time of pre-test (*M*_1_ = 18.692, *M*_2_ = 22.054, *M*_3_ = 24.550), and after three weeks of dCBT-i, the flow of the intervention group increased significantly, showing more investment and focus on learning. The flow of the other two groups also increased slightly, although these did not reach a significant level, illustrating the college students’ involvement in their studies after the beginning of school. This result was also in line with the findings of a previous study [30]. However, the study in question examined the relationship between having afternoon naps and flow. There are hardly any studies showing that dCBT-i improves the flow experience; hence our study provides important evidence and implications.

Our research is the first large dCBT-i randomized controlled trial to use healthy people as controls to exclude the influence of common life events, thus making the experiment more scientific and rigorous. We are not only concerned with the indicators of insomnia but also the improvement of daily life quality and spiritual pleasure brought about by dCBT-i. The comparison between the regular education and intervention groups shows that sleep articles can also improve sleep, but the spillover effect of dCBT-i is unique. Flow, fatigue, and cognitive flexibility affect learning, work efficiency, degree of involvement, and performance. Follow-up research should focus on work efficiency and learning adaptation that are more ecologically valid, closer to real-life situations, and consider whether some physiological indicators could be improved by dCBT-i.

However, our study is not without limitations. First, female participants constituted the majority (76.3%), which led to uneven gender distribution of participants. However, insomnia is more common among women, who are more likely to seek help. Surveys show that 60% of participants with insomnia are female patients [74]. Second, this study examined only the short-term effects of dCBT-i, and the persistence of its spillover effect is not yet clear. In the future, it is hoped that participants will be followed up in the long term to determine the stability of this effect. Third, the effects of drug treatment and caffeine use were excluded. The effects of dCBT-i on sleeping aids, refreshing substances, and frequency of use should be considered in follow-up studies.

## 5. Conclusions

This study expands the research on the effects of dCBT-i on insomnia. The results indicated that dCBT-i not only improved the quality of sleep for adults, it also relieved fatigue, and increased flow levels and cognitive flexibility. From a practical perspective, providing dCBT-i to people with poor sleep can not only improve sleep quality, but also provide learning, life, and mental health benefits. Future research should pay attention to other spillover effects, such as work efficiency, the use of sleeping pills, and the durability and stability of such effects.

## Figures and Tables

**Table 1:** The components of dCBT-i *.

Component	Explanation
Sleep hygiene education	Exercise regularly, eat regularly, and do not go to bed on an empty stomach. Make sure the bedroom is comfortable and free from light and sound. Avoid excessive beverages at night, avoid alcohol and smoking, and reduce caffeine intake. Avoid naps during the day and so on.
Stimulus control	Reduce waking time while in bed. Recreate the positive connection between drowsiness and the bed. Go to bed only when you are drowsy at night or when it is time to sleep. If the sequence fails to fall asleep, leave the bedroom for some relaxation activities.
Sleep restriction	Shorten the time spent awake in bed and increase the drive to fall asleep to improve sleep efficiency. Gradually increase your time in bed as your effective sleep time increases.
Relaxation	Muscle relaxation, breathing relaxation, imagery training, mindfulness relaxation, enhancing the control of the brain over the nervous system, reducing anxiety, relieving tension and other emotions, so that you can relax from the stress of the day and improve sleep quality.
Cognitive therapy	Correct unrealistic sleep expectations; keep falling asleep naturally, avoid over-focusing and trying to fall asleep; do not worry about losing control over your sleep; do not associate nighttime dreams with adverse daytime outcomes; Frustration arises; develop tolerance to the effects of insomnia, and do not compensate for lack of sleep at night and sleep more during the day.

* dCBT-i = digital cognitive behavioral therapy for insomnia.

**Table 2:** Demographic data of participants at baseline.

	Intervention Groups (*n* = 39)	Conventional Education Groups (*n* = 37)	Healthy Controls (*n* = 20)
Mean age (years) *	20.56 ± 1.79	21.38 ± 2.13	20.90 ± 1.41
Sex *			
Male *	30 (76.9)	28 (75.7)	15 (75.0)
Female *	9 (23.1)	9 (24.3)	5 (25.0)
Education level *			
Undergraduate *	32 (82.1)	19 (51.4)	15 (75.0)
Postgraduate *	7 (17.9)	18 (48.6)	5 (25.0)
Yearly income *			
<50,000 RMB *	8 (20.5)	9 (24.3)	6 (30.0)
50,000–100,000 RMB *	16 (41.0)	8 (21.6)	4 (20.0)
100,000–200,000 RMB *	9 (23.1)	14 (37.8)	5 (25.0)
200,000–400,000 RMB *	4 (10.3)	5 (13.5)	5 (25.0)
>400,000 RMB *	2 (5.1)	1 (2.7)	0 (0)

* Data are mean (SD) or *n* (%).

## Data Availability

The data presented in this study are available on request from the corresponding author.
